# Changes in expression of cartilaginous genes during chondrogenesis of Wharton’s jelly mesenchymal stem cells on three-dimensional biodegradable poly(L-lactide-co-glycolide) scaffolds

**DOI:** 10.1186/s11658-016-0012-2

**Published:** 2016-08-24

**Authors:** Piotr Paduszyński, Ewelina Aleksander-Konert, Alicja Zajdel, Adam Wilczok, Katarzyna Jelonek, Andrzej Witek, Zofia Dzierżewicz

**Affiliations:** 1 Department of Biopharmacy, School of Pharmacy with the Division of Laboratory Medicine in Sosnowiec, Medical University of Silesia, Katowice, Poland; 2 Centre of Polymer and Carbon Materials, Polish Academy of Sciences, Zabrze, Poland; 3 Department of Gynecology and Obstetrics, School of Medicine in Katowice, Medical University of Silesia, Katowice, Poland; 4 Department of Health Care, Silesian Medical College, Katowice, Poland

**Keywords:** Mesenchymal stem cells, Wharton’s Jelly, Umbilical cord, Biodegradable scaffold, PLGA, Chondrogenesis, Chondrocytes, Gene expression

## Abstract

**Background:**

In cartilage tissue regeneration, it is important to develop biodegradable scaffolds that provide a structural and logistic template for three-dimensional cultures of chondrocytes. In this study, we evaluated changes in expression of cartilaginous genes during in vitro chondrogenic differentiation of WJ-MSCs on PLGA scaffolds.

**Methods:**

The biocompatibility of the PLGA material was investigated using WJ-MSCs by direct and indirect contact methods according to the ISO 10993–5 standard. PLGA scaffolds were fabricated by the solvent casting/salt-leaching technique. We analyzed expression of chondrogenic genes of WJ-MSCs after a 21-day culture.

**Results:**

The results showed the biocompatibility of PLGA and confirmed the usefulness of PLGA as material for fabrication of 3D scaffolds that can be applied for WJ-MSC culture. The in vitro penetration and colonization of the scaffolds by WJ-MSCs were assessed by confocal microscopy. The increase in cell number demonstrated that scaffolds made of PLGA copolymers enabled WJ-MSC proliferation. The obtained data showed that as a result of chondrogenesis of WJ-MSCs on the PLGA scaffold the expression of the key markers collagen type II and aggrecan was increased.

**Conclusions:**

The observed changes in transcriptional activity of cartilaginous genes suggest that the PLGA scaffolds may be applied for WJ-MSC differentiation. This primary study suggests that chondrogenic capacity of WJ-MSCs cultured on the PLGA scaffolds can be useful for cell therapy of cartilage.

## Background

Articular cartilage is a tissue characterized by a limited regeneration capacity. Current clinical treatment strategies of cartilage defects do not allow for full regeneration of damaged tissue [1]. Type II collagen is the most abundant collagen in cartilage, while the most abundant proteoglycan is aggrecan. Type II collagen primarily endows the cartilage with its tensile strength, whereas aggrecan provides the osmotic resistance for cartilage to resist compressive loads. Type I and type III collagens are expressed in fibrocartilage and degenerating cartilage. Moreover, in vitro cultures of chondrocytes undergo a dedifferentiation process, gradually changing their morphology to a fibroblast-like shape, and the production of type II collagen is replaced by the production of type I collagen typical for fibrocartilage, which as biomechanically inferior compared with physiological hyaline cartilage [2, 3]. It is crucial for cartilage tissue engineering to develop biodegradable scaffolds that provide a structural and logistic template for three-dimensional (3D) cultures of chondrocytes [4]. If possible, the scaffold should have directed and controlled degradation, promote cell viability, differentiation, and extracellular matrix (ECM) production, allow for the diffusion of nutrients and waste products, and provide mechanical integrity depending on the defect size and location [5]. Aliphatic polyesters such as polylactide (PLA), polyglycolide (PGA), poly-ɛ-caprolactone and its copolymers can degrade in contact with living tissue or under the influence of external factors. Biodegradation occurs as a result of their hydrolysis to lactic acid and glycolic acid [6, 7]. Homopolymers themselves are not optimum materials for forming the 3D scaffolds for cartilage tissue regeneration. PGA is characterized by very hydrophilic properties, resulting in rapid degradation. In contrast, the degradation process of PLA is too slow. Copolymers of PLA and PGA can be easily optimized for mechanical and degradation properties by optimizing their synthesis conditions and applying appropriate polymerization initiators [8].

PLGA has proved to be an excellent material for cartilage tissue engineering due to its biodegradable properties, mechanical strength, and ease of fabrication into a considerably complex formation. Moreover, PLGA is a synthetic material that has excellent biocompatibility [6, 9, 10].

An important stimulus for the development of research in the field of tissue engineering is to find sources of stem cells, capable of being differentiated towards a specific cell type. Mesenchymal stem cells (MSCs) due to their superior proliferative and differentiation capacities offer an attractive source [9]. While the most thoroughly characterized populations of MSCs are bone marrow-derived stem cells (BMSCs) or adipose mesenchymal stem cells (ADSCs), Wharton’s jelly mesenchymal stem cells (WJ-MSCs) have drawn attention as they can easily be obtained in large quantities and show characteristics comparable to BMSCs or ADSCs [5, 11]. It has been reported that WJ-MSCs may in vitro differentiate towards chondrocytes, osteoblasts, cardiac muscle cells, skeletal muscle cells, adipocytes, β cells in the islets of Langerhans or endothelial cells [12].

The aim of this study was to evaluate the changes in transcriptional activity of genes related to hyaline cartilage and fibrocartilage during chondrogenesis of WJ-MSCs on copolymer poly (L-lactide-co-glycolide) (PLGA) scaffolds for feasibility to recover cartilage structures in vitro. Chondrocytes were used as a reference material during analyses of the gene expression pattern of differentiated cells.

## Methods

### Isolation, culture and phenotype characterization of WJ-MSCs

Isolation of WJ-MSCs was carried out using the explant method [13]. Human umbilical cord samples (*n* = 7) were collected following full term deliveries at the Department of Gynecology and Obstetrics of the Medical University of Silesia, Katowice, Poland (Central Clinical Hospital in Katowice). The patients were informed and agreed on the sampling and the purposes, and the Bioethical Committee of the Medical University of Silesia in Katowice approved the use of the samples for this research. WJ-MSCs were cultured under standard conditions (humidified atmosphere with 5 % CO_2_ at 37 °C), in a growth medium containing α-modified Minimum Essential Medium Eagle with L-glutamine and sodium bicarbonate (α-MEM; Sigma-Aldrich) supplemented with 20 % fetal bovine serum (FBS; Life Technologies), penicillin (100 U/ml; Sigma Aldrich), streptomycin (100 μg/ml; Sigma Aldrich) and 1 % Non-Essential Amino Acid Solution (NEAA; Sigma Aldrich).

Flow cytometry immunophenotypic analysis of WJ-MSCs from the third passage was performed using a Human MSC Analysis Kit (BD Biosciences) according to the manufacturer’s protocol. This kit includes the panel of antibodies for detecting cell surface markers for the minimal identification of human MSCs, which was proposed by the International Society for Cellular Therapy. The kit contained an MSC-positive cocktail of antibodies: fluorescein isothiocyanate (FITC) mouse anti-human CD90, peridinin chlorophyll protein-cyanine dye (PerCP-Cy5.5) mouse anti-human CD105, allophycocyanin (APC) mouse anti-human CD73 and negative MSC cocktail phycoerythrin (PE) conjugated antibodies (PE mouse anti-human CD45, PE mouse anti-human CD34, PE mouse anti-human CD11b, PE mouse anti-human CD19, and PE mouse anti-human HLA-DR). A BD FACSAria II flow cytometer with FACS Diva Software 6.1.2 was used in this research. A commercial line of WJ-MSCs (PromoCell) from the third passage was used for a comparison.

### Chondrocyte culture

Normal human articular chondrocytes from the hyaline cartilage of the knee were purchased from Lonza. Chondrocytes were cultured in growth medium containing α-MEM (Sigma-Aldrich), FBS (10 %; Life Technologies), penicillin (100 U/ml; Sigma Aldrich), streptomycin (100 μg/ml; Sigma Aldrich) and NEAA (1 %; Sigma Aldrich). Chondrocytes from the third passage were used in these experiments.

### PLGA biocompatibility

The biocompatibility of PLGA (molar ratio of 85:15) was tested in vitro using WJ-MSCs (seeding density: 2.5×10^4^ cells/cm^2^) by direct and indirect contact methods according to the ISO standard (ISO 10993–5) [14]. PLGA discs of 5 mm diameter were obtained by molding using methylene chloride and by molding under pressure. The cytotoxicity assessment of PLGA was performed using ultra-high molecular weight polyethylene (UHMWPE), which is a nontoxic material, and polyvinyl chloride (PVC) for a positive control. In the indirect contact test, copolymer and positive/negative control discs were placed in inserts with a pore diameter of 0.4 μm (SPL Life Sciences). In direct contact tests, either the PLGA discs, the negative control UHMWPE discs or the positive control PVC discs were placed in the center of the wells. Each experiment was performed in triplicate.

After cell exposure to the disc the viability of WJ-MSCs was assessed at 72 h and 120 h with In Vitro Toxicology Assay Kit, XTT based (TOX-2; Sigma Aldrich) according to the manufacturer’s protocol. This test provides a spectrophotometric method for estimating cell number based on the mitochondrial activity in living cells. A key component of this test is the sodium salt of XTT (2,3-bis[2-methoxy-4-nitro-5-sulfophenyl]-2H-tetrazolium-5-carboxyanilide inner salt), which is reduced by mitochondrial dehydrogenases of viable cells. Reduction of the tetrazolium ring of XTT yields an orange formazan derivative, which is water soluble and is measured spectrophotometrically. An increase or decrease in viable cells relative to control cells results in a change in the amount of formazan formed, indicating the degree of cytotoxicity caused by the tested material [15].

### Fabrication of scaffold

In this study we used scaffolds fabricated from PLGA. A copolymer of poly(L-lactide-co-glycolide) in a molar ratio of 85:15 was synthesized using zirconium acetylacetonate – a non-toxic initiator of polymerization. The copolymer was characterized by the number average molecular weight (M_n_) of 50 kDa and the weight average molecular weight (M_w_) of 105 kDa. Scaffolds were fabricated by the solvent casting/salt-leaching technique as described by Pamuła et al. in the Centre of Polymer and Carbon Materials (Polish Academy of Sciences) [16]. Microstructure of obtained PLGA scaffolds was analyzed by a scanning electron microscope (Quanta 250 FEG, FEI Company; accelerating voltage 5 kV during scanning), a confocal laser scanning microscope (Olympus FluoView), and an inverted phase contrast microscope (Nikon Eclipse TS100).

### Invasion of the PLGA scaffolds by WJ-MSCs

WJ-MSCs (seeding density: 5×10^6^ cells per scaffold) were loaded into the pores of PLGA scaffolds in a growth medium containing α-modified Minimum Essential Medium Eagle with L-glutamine and sodium bicarbonate (α-MEM; Sigma-Aldrich) supplemented with 20 % fetal bovine serum (FBS; Life Technologies), penicillin (100 U/ml; Sigma Aldrich), streptomycin (100 μg/ml; Sigma Aldrich) and 1 % Non-Essential Amino Acid Solution (NEAA; Sigma Aldrich). To observe the effects of seeding, the cells were stained with CellLight Tubulin-GFP BacMan 2.0 (Life Technologies) reagent according to the manufacturer’s protocol. The reagent labels tubulin with green fluorescent protein (GFP) in live cells. The invasion of the scaffolds by WJ-MSCs was assessed at days 1, 7, and 14 of culture in the growth medium, using a confocal laser scanning microscope (Olympus FluoView).

### In vitro chondrogenesis

WJ-MSCs and chondrocytes from the third passage were used for all chondrogenic experiments. Cells were grown in 2D cell culture dishes in growth medium (seeding density: 5×10^3^ cells/cm^2^ for WJ-MSCs and 1×10^4^ cells/cm^2^ for chondrocytes) and chondrogenic medium (seeding density: 2.5×10^4^ cells/cm^2^ for WJ-MSCs and chondrocytes). Cells were also grown on 3D PLGA scaffolds in growth medium (seeding density: 5×10^6^ cells per scaffold for WJ-MSCs and chondrocytes) and chondrogenic medium (seeding density: 5×10^6^ cells per scaffold for WJ-MSCs and chondrocytes). Chondrogenesis was induced by a chondrogenic medium containing: α-MEM (Sigma Aldrich), dexamethasone (100 nM; Sigma Aldrich), TGF-β3 (10 ng/ml; Gene Way), ITS+ Premix (1 %; BD Biosciences), ascorbic acid 2-phosphate (50 μg/ml; Sigma Aldrich), FBS (2 %; Life Technologies), NEAA (1 %; Sigma-Aldrich), penicillin (100 U/ml; Sigma Aldrich), streptomycin (100 μg/ml; Sigma Aldrich) and sodium pyruvate (1 %; Life Technologies) [17, 18]. All the cultures were grown for 21 days. Finally, after this time period total RNA was isolated from all cultures and real-time PCR analysis was performed. The calibrators (controls) used to analyze the relative changes in gene expression for the 2D environment were WJ-MSCs and chondrocytes respectively, which were grown in cell culture dishes (2D) in growth medium. For the 3D culture system the calibrators (controls) used to analyze the relative changes in gene expression were WJ-MSCs and chondrocytes respectively, which were grown on PLGA scaffolds in growth medium.

### RNA isolation and real-time PCR analysis

The total RNA was extracted using the RNeasy Mini Kit (Qiagen), according to the manufacturer’s protocol. To remove possible contamination of DNA, samples were treated with DNase I solution. The amount of isolated RNA was checked using the Quant-IT RiboGreen RNA Reagent Kit (Life Technologies). Fluorometric measurement results allowed the determination of the starting amount of samples for real-time PCR.

Real-time PCR was performed in one step using the Power SYBR Green RNA-to-Ct 1-Step Kit (Applied Biosystems). This reaction was carried out using primers specific to a given sequence (Table 1). The expression of the following genes was examined: collagen type I, collagen type II, collagen type III and aggrecan. Glyceraldehyde-3-phosphate dehydrogenase (GAPDH) was used as a reference gene. The reaction conditions were as follows: an initial denaturation (95 °C for 5 min), polymerase activation (95 °C for 10 min) followed by 39 cycles of denaturation (95 °C for 15 s), annealing (60 °C for 30 s), and extension (72 °C for 30 s). To ensure reliability of the results obtained, each sample was analyzed in triplicate. Quality of the PCR products was checked by melting curve analysis. To calculate the differences in transcriptional activity of the analyzed genes between treatment and control samples (calibrator), the ΔΔC_T_ method was used. Determining gene expression profiles and statistical analysis was performed using REST 2009 software (Qiagen).

**Table 1:** Sequences of primers used for real-time PCR

Gene	Primer sequences	Accession no.	Amplicon length (bp)
GAPDH	5’ GAAGGTGAAGGTCGGAGTC 3’	NM_002046.3	226
	5’ GAAGATGGTGATGGGATTTC 3’		
Collagen type I	5’ CCACCAATCACCTGCGTACA 3’	NM_000088	119
	5’ CATCGCACAACACCTTGCC 3’		
Collagen type II	5’ TGCTGACGCTGCTCGTCGC 3’	NM_001844.4	163
	5’ TCGTCGCAGAGGACAGTCCCA 3’		
Collagen type III	5’ CAGCAGGGTGCAATCGGCAGT 3’	NM_000090	177
	5’ TGGTTGCCCTGGGTGGCCT 3’		
Aggrecan	5’ CAAGAGCAGTGCAATCGTTGG 3’	NM_001135.2	127
		NM_013227.2	
	5’ ACATTCAGCTGCGGTTCCG 3’		

### Statistical analysis

The results of the experiment evaluating the biocompatibility of PLGA material were checked for normality with the Shapiro*-*Wilk test. The statistical differences between groups were calculated using one-way analysis of variance (ANOVA). To determine the homogeneity of variance the Brown-Forsyth test was used. To assess statistically significant differences between the means an LSD (least significant difference) test was performed (StatSoft, Inc. 2011, STATISTICA version 10). Relative gene expression analyses were performed using the software REST 2009 (Qiagen). Statistical significance was declared at *p* < 0.05.

## Results

### WJ-MSC culture and phenotype characterization

The cells that had migrated from the explants were able to adhere and proliferate on the surface of the polystyrene culture dishes, which is characteristic for MSCs. The obtained cell lines of WJ-MSCs were able to grow in vitro in culture for a number of passages. Cells were cultured using the initial density range of 3×10^3^-5×10^3^ cells/cm^2^. WJ-MSCs reached confluence in culture vessels after 5–7 days of incubation under standard conditions.

**Fig. 1:**
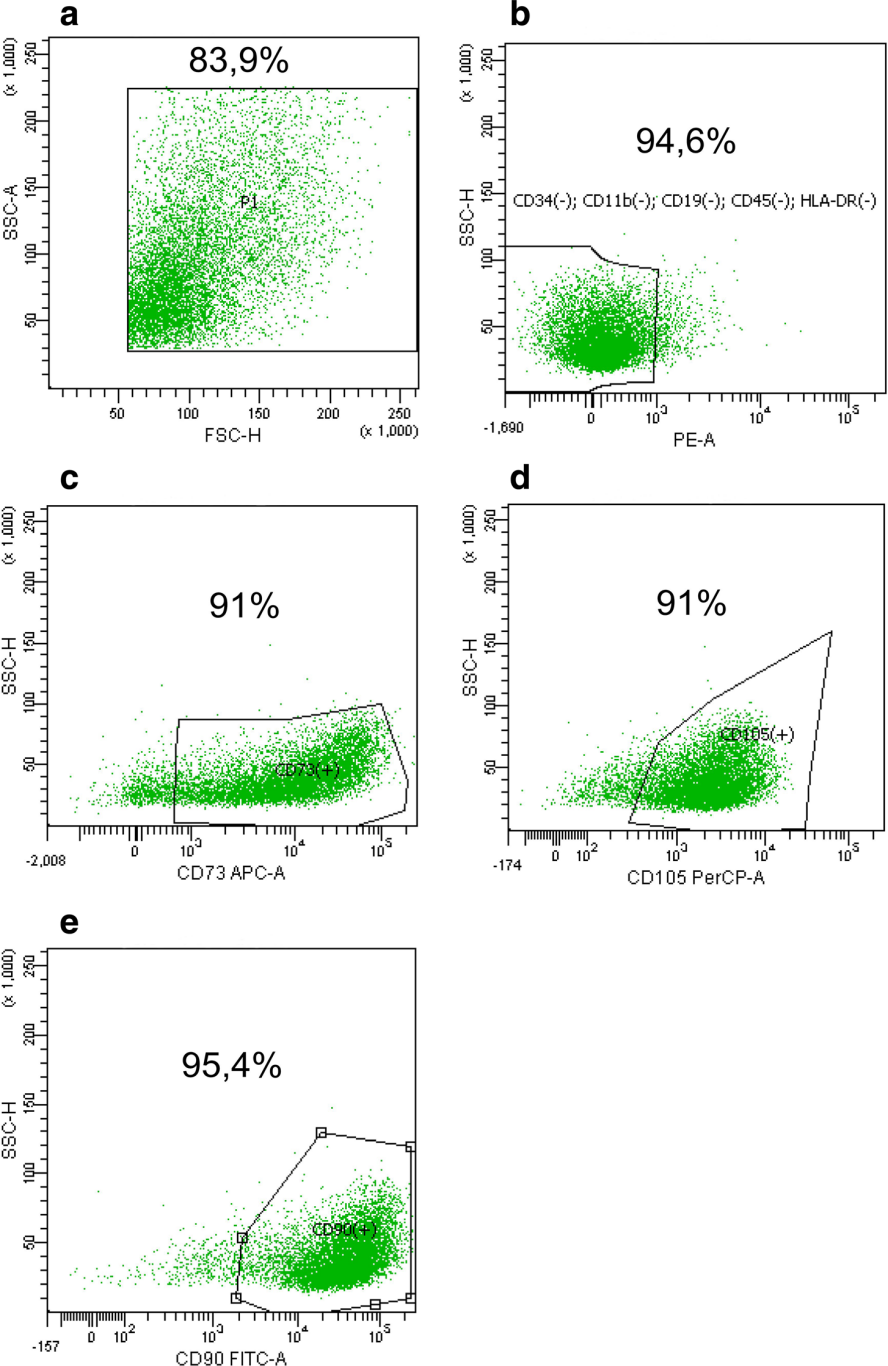
Scattergrams representing the profile of isolated WJ-MSC samples analyzed by flow cytometry evaluating the cell surface markers. **a** Dot plot presenting data from forward scatter (FSC) and side scatter (SSC) detectors and percentage of living cells; **b** dot plot presenting fluorescence signal from negative cocktail of PE conjugated antibodies (PE CD45, PE CD34, PE CD11b, PE CD19 and PE HLA-DR) and data from SSC; **c** dot plot presenting fluorescence signal from APC-CD73 antibodies and data from SSC; **d** dot plot presenting fluorescence signal from PerCP-Cy 5.5-CD105 antibodies and data from SSC; **e** dot plot presenting FITC CD90 antibody fluorescence signal and data from SSC; in the plot in panel B the percentage of negative cells for hematopoietic markers and in the plots in panels C-E the percentage of positive cells for certain MSC markers are shown

Immunophenotypic analysis of WJ-MSCs by flow cytometry revealed cells with high expression of CD73 (91 % of the cells; Fig. 1c), CD105 (91 % of the cells; Fig. 1d) and CD90 (95.4 % of the cells; Fig. 1e) markers and absent expression of hematopoietic markers (94.6 % of the cells) such as CD45, CD34, CD11b, CD19 and HLA-DR (Fig. 1b).

### PLGA biocompatibility

**Fig. 2:**
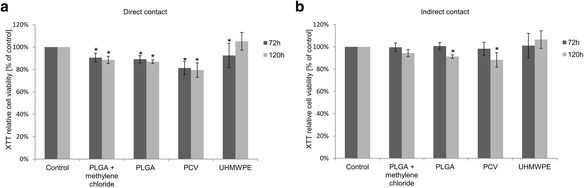
Viability of WJ-MSCs evaluated using two methods: **a** – direct contact, **b** – indirect contact with PLGA biomaterial and reference materials after 72 h and 120 h of culture, expressed as a percentage of control (100 %); mean ± standard deviation; * *p* < 0.05 (ANOVA with *post hoc* LSD test), PVC – polyvinyl chloride, PLGA – poly(L-lactide-co-glycolide); UHMWPE – ultra-high molecular weight polyethylene

The results of the direct contact test, after 72 h of WJ-MSCs’ exposure to PLGA discs obtained by molding using methylene chloride, show a 10 % decrease in viability of WJ-MSCs compared to the control. A similar effect was observed in cultures with the PLGA discs obtained by molding under pressure. In the case of the PVC discs (positive control), an approximately 20 % decrease in cell viability compared with that in the control was observed. The negative control, where cells were treated with UHMWPE, was also characterized by a slight reduction (8 %) of viability relative to the control (Fig. 2a). After 120 h of culture of WJ-MSCs with PLGA discs obtained by molding using methylene chloride, we observed slight inhibition (11 %) of the metabolic activity of cells in comparison to control cells. Also WJ-MSCs cultured with PLGA discs obtained by molding under pressure showed a 13 % decrease of viability in comparison to control culture. WJ-MSC exposure to PVC discs was characterized by the largest reduction (21 %) in cell metabolic activity compared to controls. The changes of WJ-MSCs’ viability in culture with UHMWPE discs were not statistically significant (Fig. 2a). The obtained results demonstrated that the PLGA copolymer is a highly biocompatible material. Inhibition of growth in the experimental groups was not more than 30 %, relative to the control culture, which, according to ISO 10993–5, is the limit for biocompatible materials in cell cultures in vitro.

To assess the biocompatibility of PLGA in relation to WJ-MSCs the indirect contact test with inserts (Fig. 2b) was used. After 72 h of WJ-MSCs’ exposure to the PLGA discs (obtained by molding under pressure and using methylene chloride) determination of cell proliferation showed no differences compared to the control. The viability of WJ-MSCs in cultures which were positive or negative controls showed no difference compared with that in the control. After 120 h of cultivation, the XTT results showed that the cultures with PLGA discs, produced by using methylene chloride, and by molding under pressure, were characterized by slight inhibition of proliferation (about 6 % and 9 % respectively) compared to the control. The highest inhibition of metabolic activity of the cells was observed in the negative control cultures. Viability determination of WJ-MSCs in cultures with UHMWPE showed no difference compared to the control culture. The results from indirect contact tests showed biocompatibility of PLGA in accordance with the guidelines of ISO 10993–5. The obtained results confirmed the usefulness of PLGA as material for fabrication of 3D scaffolds that can be applied for WJ-MSC culture. Similar analyses were carried out using chondrocytes and fibroblasts, where biocompatibility of PLGA was confirmed (data not shown).

### Characteristics of PLGA scaffolds

**Fig. 3:**
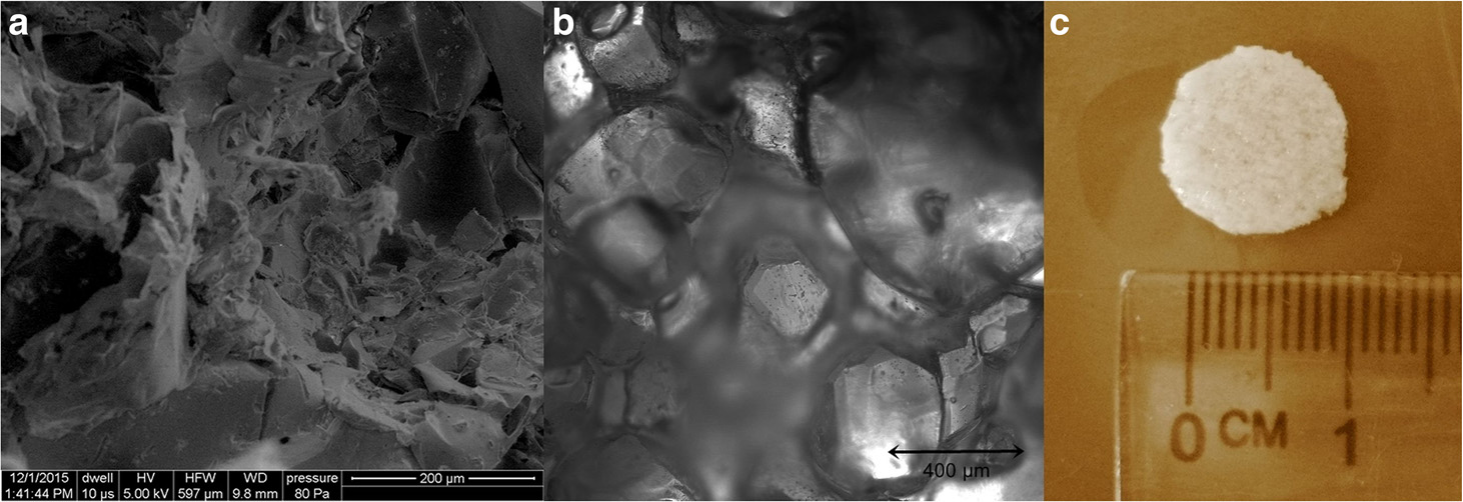
Microstructure and macrostructure of PLGA scaffolds: **a** scanning electron micrographs of the cross section (Quanta 250 FEG; FEI Company), **b** image from confocal laser scanning microscope (Olympus FluoView), **c** morphology of representative PLGA scaffold

The obtained PLGA scaffolds were characterized by the following parameters: density of polymer (ρ_p_) = 1.0063 g/cm^3^, volume of water in pores of the scaffold (V_pores_) = 0.24644 cm^3^, volume of polymer (V_polymer_) = 0.2404 cm^3^, porosity of scaffold (P_0_) = 50.7 %, and absorbency of scaffold (A) = 50.4 %. Analysis of these parameters showed that the obtained scaffolds made of PLGA were characterized by a porous structure with adequate stiffness and absorbency. High rigidity of the PLGA scaffolds can provide adequate mechanical properties and can retain high stability, even during long*-*term cell culture*.* The obtained scaffolds have sufficient porosity, and their pore size provides high water permeability in in vitro cultures. The analysis of the microstructure of PLGA scaffolds by scanning electron, confocal laser scanning and inverted phase contrast microscopy (Fig. 3) showed the pore size in the range of 200–500 μm.

### WJ-MSC growth on PLGA scaffolds

**Fig. 4:**
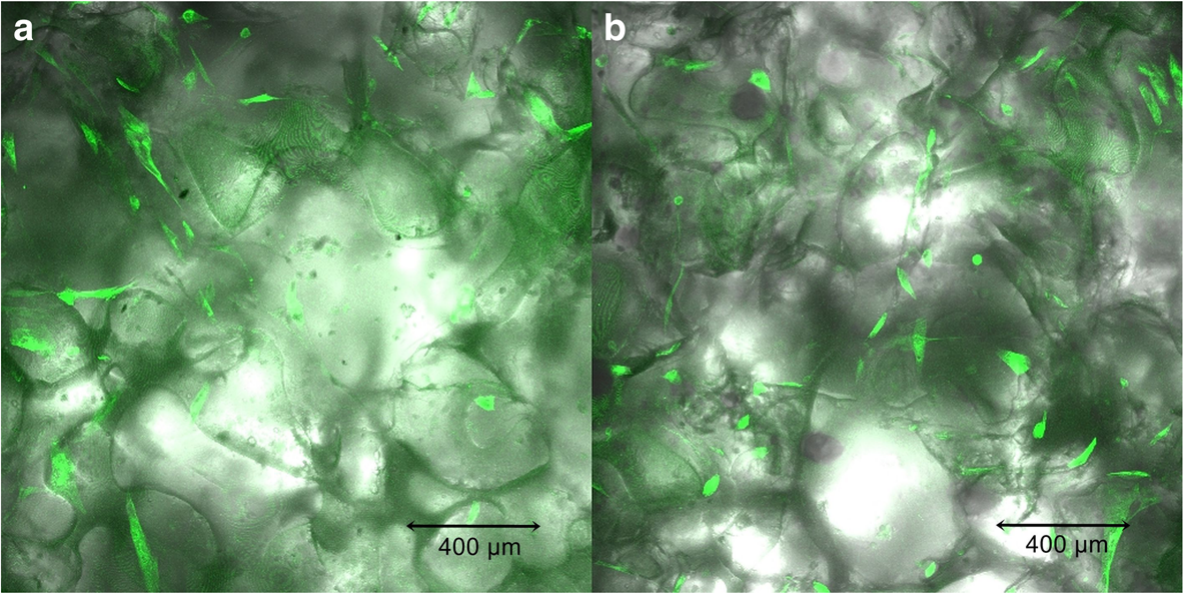
WJ-MSC invasion of PLGA scaffolds after 14 days of culture in: **a** – upper and **b** – bottom surface (confocal laser scanning microscope Olympus FluoView, magnification 100x)

The microscopic analysis of WJ-MSC invasion in 7-day culture showed an increased number of cells in both upper and bottom surfaces of the scaffold. The depth of invasion of WJ-MSCs was estimated to be 100–120 μm. The largest numbers of cells in the bottom and upper surfaces of the PLGA scaffolds were observed after 14 days of culture (Fig. 4). The cells attached to the scaffolds were spindle-shaped. The increase in cell number demonstrated that scaffolds made of PLGA copolymers enabled WJ-MSCs proliferation. The highest density of WJ-MSCs occurred in the upper and lower surfaces of PLGA scaffolds. The cells also migrated to the middle regions and colonized the interior pores to a depth of 120 μm.

### Gene expression

**Fig. 5:**
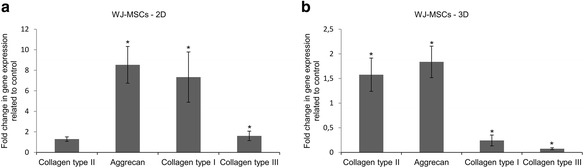
Transcriptional activity of collagen type II, aggrecan, collagen type I, and collagen type III genes expressed as fold change in relation to control (expression = 1) in WJ-MSCs cultured for 21 days on: **a** 2D – monolayer, **b** 3D PLGA scaffold. Control culture was carried out in growth medium in: **a** 2D – monolayer, **b** PLGA scaffold. Growth medium contained inter alia α-MEM medium with 20 % FBS. Chondrogenic medium contained inter alia differentiation factors such as dexamethasone and TGFβ3, ascorbic acid 2-phosphate, ITS + Premix, sodium pyruvate and 2 % FBS. * *p* < 0.05

As shown in Fig. 5a, WJ-MSCs cultured in the chondrogenic medium in 2D culture were characterized by a 1.3-fold increase in the expression of collagen type II when compared to the 2D culture in growth medium. WJ-MSCs cultured in chondrogenic medium showed a significant increase in aggrecan expression, approximately 8.53-fold higher than in 2D culture after 21 days of culture in growth medium. Transcriptional activity of collagen type I and type III in 2D culture of WJ-MSCs in chondrogenic medium significantly increased about 7.4-fold and 1.6-fold respectively in comparison to 2D culture in growth medium. In contrast, WJ-MSCs cultured on PLGA scaffold in chondrogenic medium were characterized by 1.6-fold increased expression of a key marker of chondrogenesis – collagen type II – compared to PLGA scaffold culture in growth medium (Fig. 5b). The expression of aggrecan was approximately 1.8-fold higher in PLGA scaffold culture in chondrogenic medium compared to PLGA scaffold culture in growth medium. In contrast, the analysis of transcriptional activity of collagen type I and type III in WJ-MSCs cultured on PLGA scaffold in differentiation medium showed significantly decreased expression of these genes, approximately 4.13-fold and 13.5-fold respectively compared to WJ-MSC culture on PLGA scaffold in growth medium.

**Fig. 6:**
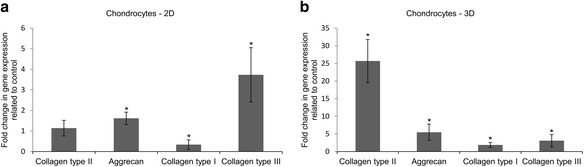
Transcriptional activity of collagen type II, aggrecan, collagen type I, and collagen type III genes expressed as fold change in relation to control (expression = 1) in chondrocytes cultured for 21 days on: **a** 2D – monolayer, **b** 3D – PLGA scaffold. Control culture was carried out in growth medium in: **a** 2D – monolayer, **b** PLGA scaffold. Growth medium contained inter alia α-MEM medium with 10 % FBS. Chondrogenic medium contained inter alia differentiation factors such as dexamethasone and TGFβ3, ascorbic acid 2-phosphate, ITS + Premix, sodium pyruvate and 2 % FBS. * *p* < 0.05

As shown in Fig. 6a, chondrocytes in 2D culture in chondrogenic medium showed minor changes in expression of collagen type II in comparison to 2D culture of chondrocytes in growth medium. Chondrocytes in 2D culture exhibited 1.6-fold higher expression of the aggrecan gene than in 2D culture of chondrocytes in growth medium. Collagen type I in 2D culture of chondrocytes in chondrogenic medium was characterized by 2.94-fold decreased expression in comparison to 2D culture of chondrocytes in growth medium. In contrast, expression of collagen type III in chondrocytes cultured in a 2D environment was 3.7-fold higher than in the chondrocyte 2D culture in growth medium. When chondrocytes were grown for 21 days on PLGA scaffold in chondrogenic medium, the transcriptional activity of collagen type II significantly increased, about 25.7-fold higher than in PLGA scaffold culture of chondrocytes in growth medium (Fig. 6b). Another marker of chondrogenesis – aggrecan – was also upregulated in culture of chondrocytes on PLGA scaffold in chondrogenic medium. Expression of this gene was 5.5-fold higher in comparison to culture of chondrocytes on PLGA scaffold in growth medium. Transcriptional activity of collagen type I and type III in culture of chondrocytes in PLGA scaffold in chondrogenic medium increased about 1.9-fold and 3.1-fold respectively in comparison to chondrocytes which were grown in growth medium on PLGA scaffold.

## Discussion

Successful cartilage tissue engineering requires cells capable of undergoing chondrogenic differentiation upon treatment with appropriate biochemical and physical regulatory factors and a 3D scaffold which can provide the cell adhesion, proliferation, differentiation and cartilage-specific ECM formation. MSCs can be easily obtained and cultured for multiple passages without losing differentiation potential. Among MSCs, BMSCs and ADSCs are most often used for cartilage tissue engineering in vitro and in vivo [11, 19, 20], but their application in humans is still in the early stages. So far BMSCs and ADSCs have been compared in both pellet cultures and other 3D culture systems such as scaffolds, and the results indicated lower chondrogenic potential for the adipose tissue derived MSCs than for bone marrow-derived MSCs [11, 20, 21]. Extra-embryonic tissue-derived MSCs have multipotent characteristics and proliferate faster than adult MSCs. Moreover, these cells can be isolated and used without ethical problems, because extra-embryonic tissues are normally discarded after birth [22]. Therefore, in the present study we decided to assess the feasibility of WJ-MSC differentiation towards chondrocytes on PLGA scaffolds.

The possibility of application of WJ-MSCs in the treatment of cartilage defects results from their relevant biological properties, in particular the capacity to generate fully differentiated, specialized cells of the body including the cartilage cells, immune immaturity, acceptable transplantation in partial HLA incompatibility, low probability of infection transmission and prolonged self-renewal [22, 23]. In the present work, the WJ-MSCs isolated from umbilical cord were analyzed by flow cytometry to determine their phenotype. Immunophenotypic analysis of WJ-MSCs revealed MSCs with high expression of human MSCs markers and absent expression of hematopoietic protein on the surface. Moreover, the PLGA biomaterial used in the present experiment showed high biocompatibility and no impact of any residual solvents on the growth of WJ-MSCs.

The key objective was to evaluate WJ-MSC chondrogenic gene expression in two different systems: a 3D cell culture system and a 2D culture. Analysis of changes in expression of genes related to chondrogenesis of WJ-MSCs over a 21-day culture showed an increase in the transcriptional activity of collagen type II in PLGA 3D scaffold systems and in 2D culture. Another marker of hyaline cartilage, aggrecan, was also upregulated both in 2D culture and PLGA scaffold in chondrogenic medium. This may suggest that WJ-MSCs differentiated into cartilage cells. Transcriptional level of collagen type I decreased markedly in the PLGA scaffold but increased significantly in 2D culture. The expression of another marker of fibrocartilage, collagen type III, decreased significantly in culture on PLGA scaffold, but increased in 2D culture. On this basis it can be concluded that PLGA scaffolds with relevant differentiating factors provide conditions for proper WJ-MSC chondrogenesis in vitro. The results of the experiment on chondrocytes showed high expression of hyaline cartilage markers – collagen type II and aggrecan – in the scaffold culture. The observed changes in the level of collagen type II and aggrecan mRNA – markers of hyaline cartilage – suggest that the PLGA scaffolds consisting of PLA and PGA (85 : 15) may be applied for WJ-MSC chondrogenic differentiation. Additionally, during the experiments the WJ-MSC transdifferentiation was checked. Staining with alizarin s (osteogenic transdifferentiation) and oil red (transdifferentiation into adipocytes) gave negative results in every case. The chondrogenic capacity of WJ-MSCs was also checked by alcian blue staining or safranin O staining. In this case the results were always positive.

Wang et al. also demonstrated that PGA scaffolds facilitated chondrogenesis of WJ-MSCs [24]. Chondrogenic differentiation of WJ-MSCs can be enhanced when cultured on nanofibrous substrates with a sequential two culture medium environment. The differentiated WJ-MSCs were able to upregulate the production of hyaluronic acid, glycosaminoglycans (GAGs) and expression of SOX9, COMP, collagen type II, FMOD genes [25], which is in agreement with some of our observations.

Scaffolds provide the space available for tissue to develop and the physical support for cell growth. Use of 3D scaffolds to support and maintain contact between cells and biochemical factors essential for differentiation is one of the main applications of chondrogenic differentiation of MSCs. In addition to the size of the pores, the morphology can significantly influence the performance of an implanted matrix and the rate of growth of a tissue. Cell morphology together with cell activity, expression of selected genes and ECM production are closely connected with macro and microstructure of scaffolds [3, 26, 27]. Pores of the scaffold facilitate invasion of PLGA by WJ-MSCs and also provide appropriately high stiffness, which additionally enhances chondrogenesis activated by bioactive factors such as TGFβ3, dexamethasone and ascorbic acid 2-phosphate [28]. In the case of chondrogenesis of WJ-MSCs on scaffolds stimulated by chondrogenic factors, a critical step is establishing cell-ECM and cell-cell interactions during migration, proliferation and differentiation. This is regulated by the connection of cell adhesion molecules of the surrounding cells, formation of gap junctions and changes in the cytoskeletal architecture [3, 29]. Structure and chemical composition of the scaffold, biochemical inducers and cell communication together activate intracellular signaling pathways which promote differentiation of WJ-MSCs into chondrocytes. The 3D environment provided by PLGA scaffolds closely cooperates with chondrogenic bioactive factors to achieve the desired chondrogenic differentiation of WJ-MSCs and chondrocytes.

The advantage of using WJ-MSCs in chondrogenesis is their better chondrogenic potential compared to MSCs derived from other sources, because their native environment is rich in hyaluronic acid, similarly as in cartilage, and like chondrocytes they are exposed to very low oxygen tension [25, 30, 31]. Chondrocytes in vivo are exposed to a low oxygen concentration and still maintain the capacity of proliferation and differentiation. Cartilage has no blood supply, deriving nutrients from the surrounding synovial fluid. MSCs in lower oxygen tension have a better chondrogenic response in cell aggregate culture, pellet culture and micromass culture, which is reflected in increased transcriptional activity of genes related to ECM. This may reflect the fact that, in vivo, articular cartilage, Wharton’s jelly and bone marrow are reported to exist within a range of 1 % to 7 % oxygen concentration. A group of transcriptional factors called hypoxia inducible factors regulates the cellular response to changing oxygen tension. The downstream targets involve several hydroxylases which are critical in formation of collagen fibers. However, lower oxygen tension is not the only factor that can influence chondrogenesis [32–34]. In our experiments with PLGA scaffolds where cells aggregate or in 2D cell cultures with high cell density, spaces of lower oxygen tension may occur, and this is probably an additional factor stimulating chondrogenesis of WJ-MSCs.

A variety of biopolymers can be used as scaffolds for cartilage tissue engineering, including poly(L-lactide)/poly(ε-caprolactone) [35], poly(ε-caprolactone) [36], and poly(lactide-coglycolide) [37]. However, these materials may be problematic because they can induce inflammation due to elevated acidity during polymer degradation [38].

## Conclusions

Analysis of the changes in gene expression of cartilaginous extracellular matrix components of WJ-MSCs cultured on PLGA scaffolds suggests that their chondrogenic capacity could be useful for cell therapy in cartilage diseases.

## Abbreviations

ADSCs, adipose mesenchymal stem cells; ANOVA, analysis of variance; APC, allophycocyanin; BMSCs, bone marrow-derived stem cells; ECM, extracellular matrix; FBS, fetal bovine serum; FITC, fluorescein isothiocyanate; FSC, forward scatter; GAGs, glycosaminoglycans; GAPDH, glyceraldehyde-3-phosphate dehydrogenase; GFP, green fluorescent protein; LSD, least significant difference; MSCs, mesenchymal stem cells; NEAA, non-essential amino acid solution; PCR, polymerase chain reaction; PE, phycoerythrin; PerCP-Cy5.5, peridinin chlorophyll protein-cyanine dye; PGA, polyglycolide; PLA, polylactide; PLGA, poly(L-lactide-co-glycolide); PVC, polyvinyl chloride; SSC, side scatter; TGF-β3, transforming growth factor β3; UHMWPE, ultra-high molecular weight polyethylene; WJ-MSCs, Wharton’s jelly mesenchymal stem cells; α-MEM, α-modified Minimum Essential Medium Eagle
